# Resveratrol potentiates effects of simvastatin on inhibition of rat ovarian theca-interstitial cells steroidogenesis

**DOI:** 10.1186/1757-2215-7-21

**Published:** 2014-02-13

**Authors:** Israel Ortega, Jesus A Villanueva, Donna H Wong, Amanda B Cress, Anna Sokalska, Scott D Stanley, Antoni J Duleba

**Affiliations:** 1 Department of Obstetrics and Gynecology, School of Medicine, University of California, Davis, CA 95616, USA; 2 IVI-Madrid, Madrid, Spain; 3 Department of Gynecology, Obstetrics and Gynecological Oncology, Karol Marcinkowski University of Medical Sciences, 60-701 Poznan, Poland; 4 Department of Obstetrics and Gynecology, University of Pennsylvania, Philadelphia, PA 19104, USA; 5 Department of Molecular Biosciences, University of California, Davis, CA 95616, USA

**Keywords:** Androgens, CYP17A1, Ovarian theca-interstitial cells, Resveratrol, Simvastatin, Steroidogenesis

## Abstract

**Background:**

Polycystic ovary syndrome (PCOS) is characterized by ovarian enlargement, hyperplastic theca compartment and increased androgen production due to, at least in part, excessive expression of several key genes involved in steroidogenesis. Previously, our group has demonstrated that simvastatin, competitive inhibitor of 3-hydroxy-3-methyl-glutaryl-CoA reductase (HMG-CoA reductase), a rate-limiting step of the mevalonate pathway, reduces rat-theca interstitial cell steroidogenesis by inhibiting *Cyp17a1* gene expression, the key enzyme of the androgen biosynthesis pathway. Recently, we demonstrated that resveratrol, a bioflavonoid abundant in red grapes, decreases rat theca-interstitial cell steroidogenesis and this suppressive effect is mediated through mechanisms independent of the mevalonate pathway. The present study evaluated the effect of combining simvastatin and resveratrol treatments on rat theca-interstitial cell steroidogenesis.

**Methods:**

Rat theca-interstitial cells isolated from 30 day-old female rats were cultured for up to 48 h with or without simvastatin (1 μM) and/or resveratrol (3-10 μM). Steroidogenic enzymes gene expression was evaluated by quantitative real time PCR and steroid levels were measured by liquid chromatography-mass spectrometry. Comparisons between groups were performed using ANOVA and Tukey test.

**Results:**

Resveratrol potentiated inhibitory effects of simvastatin on androstenedione and androsterone production in theca-interstitial cells. This suppressive effect correlated with profound inhibition in *Cyp17a1* mRNA expression in the presence of a combination of resveratrol and simvastatin.

**Conclusions:**

The present findings indicate that resveratrol potentiates the simvastatin-induced inhibitory effect on theca-interstitial cell androgen production, raising the possibility of development of novel treatments of PCOS.

## Background

Theca-interstitial cells play a prominent role in folliculogenesis, controlling follicle growth and atresia, providing mechanical support for ovarian follicles and regulating ovarian steroidogenesis [1]. Differentiation of theca-interstitial cells from steroidogenically inactive cells into androgen-producing cells occurs during preantral stages of follicular development and involves expression of steroid pathway biosynthetic genes. Under pathological conditions such as polycystic ovary syndrome (PCOS), ovaries are significantly enlarged and individual theca cells produce excessive amounts of androgens due, at least in part, to increased expression of several genes involved in steroidogenesis, including *STAR*, *CYP11A1*, *HSD3B2* and *CYP17A1*[2-4]. Among the above-listed genes, overexpression of *CYP17A1*, the key gene regulating androgen biosynthesis pathway, has been shown to play a prominent role in androgen excess in women with PCOS [5,6].

Simvastatin belongs to the family of statins, competitive inhibitors of 3-hydroxy-3-methylglutaryl coenzyme A (HMG-CoA) reductase (HMGCR), the rate-limiting step of the mevalonate pathway. Thus, the effects of statins may be related to decreased availability of several downstream products of this pathway, such as substrates of isoprenylation: farnesyl pyrophosphate (FPP) and geranylgeranyl pyrophosphate (GGPP), as well as reduction of the availability of cholesterol. In addition to the beneficial effects of statins on cardiovascular diseases due to its cholesterol-lowering action [7,8], these agents have emerged over the past decade as promising novel treatments of endocrine disorders such as PCOS due to their anti-proliferative, androgen-lowering and anti-inflammatory properties. We have demonstrated that simvastatin reduces rat theca cell proliferation by mechanisms involving inhibition of isoprenylation [9,10]. Furthermore, our recent *in vitro* study has shown that simvastatin inhibits rat theca-interstitial steroidogenesis primarily by inhibiting *Cyp17a1* mRNA expression, and this suppressive effect is mediated, at least in part, by decreased isoprenylation [10]. In clinical trials, we have shown that simvastatin treatment improves lipid profile, decreases systemic inflammation and reduces androgen levels in women with PCOS [11-13]. However, statins have potential adverse effects including a recently demonstrated risk of development of type 2 diabetes [14]. Hence, there is an urgent need to identify new agents that would either replace statins or potentiate their beneficial effects while reducing their adverse effects. We propose that resveratrol is such an agent. Notably, clinical use of resveratrol has been recently shown to reduce insulin resistance and likely decrease the risk of development of type 2 diabetes [15].

Resveratrol (*trans*-3,5,4′-trihydroxystilbene) is a natural polyphenol produced by several plants to protect them from pathogens such as bacteria and fungi. This phytoestrogen is found in grapes, nuts, berries and red wine and possesses a broad range of beneficial properties in different tissues, including anti-carcinogenic, cardioprotective, anti-inflammatory and anti-oxidant [16-19]. Previously, we found that resveratrol promotes apoptosis and inhibits proliferation in rat theca-interstitial cells, counteracting the anti-apoptotic and proliferative effects of insulin [20]. Additionally, we recently demonstrated that resveratrol reduces androgen production and *Cyp17a1* mRNA gene expression, at least partly, via inhibition of Akt/PKB phosphorylation in rat theca-interstitial cells [21].

To date, only a few studies evaluated the potential beneficial effects of combined therapy using statin in conjunction with resveratrol. Penumathsa *et al.* demonstrated that simvastatin in combination with resveratrol is more cardioprotective than simvastatin alone using an ischemic rat heart model [22]. In our recent *in vitro* studies, resveratrol potentiated simvastatin-induced inhibition of rat theca-interstitial cell proliferation [23], as well as it augmented the inhibitory effects of simvastatin on cholesterol biosynthesis and HMGCR enzyme activity in primary cultures of human endometrial stromal cells [24].

In view of these considerations, we proposed that resveratrol may enhance simvastin-induced inhibition in steroidogenesis, exerting complementary actions on mechanisms regulating both gene expression and androgen production. In the present study we evaluated the effect of combining resveratrol and simvastatin treatments on rat theca-interstitial cell steroidogenesis. We demonstrated that resveratrol potentiated inhibitory effects of simvastatin on androstenedione and androsterone production by theca-interstitial cells. This suppressive effect correlated with profound inhibition in *Cyp17a1* mRNA expression in the presence of a combination of resveratrol and simvastatin.

## Methods

### Animals

Female Sprague-Dawley rats were obtained at age 22 days from Charles River Laboratories (Wilmington, MA) and housed in an air-conditioned environment and a 12-h light/12-h dark cycle. All animals received standard rat chow and water *ad libitum*. At the age of 27, 28 and 29 days, the rats were injected with 17β-estradiol (1 mg/0.3 ml of sesame oil s.c.) to stimulate ovarian development and growth of antral follicles. Twenty-four hours after the last injection, the animals were anesthetized using ketamine and xylazine (i.p.) and euthanized by intracardiac perfusion using 0.9% saline. All treatments and procedures were carried out in accordance with accepted standards of human animal care as outlined in the National Institutes of Health Guide for the Care and Use of Laboratory Animals and a protocol approved by the Institutional Animal Care and Use Committee at the University of California, Davis.

### Cell culture and reagents

The collection and purification of ovarian theca-interstitial cells were performed as described previously [25,26]. Briefly, the ovaries were removed from the animals and dissected free of oviducts and fat under a dissecting microscope. After a 60-minute collagenase digestion, theca-interstitial cells were purified using discontinuous Percoll gradient centrifugation. The cells were counted, and viability, as assessed by the trypan blue exclusion test, was routinely in the 90%-95% range. Theca-interstitial cells were incubated for 48 hours in 24-well fibronectin-coated plates at a density of 400,000 cells/well. The cultures were carried out at 37°C in an atmosphere of 5% CO_2_ in humidified air in serum-free McCoy’s 5A culture medium supplemented with 1% antibiotic/antimycotic mix, 0.1% bovine serum albumin and 2 mM L-glutamine. The cells were incubated in the absence (control) or in the presence of simvastatin (1 μM) and/or resveratrol (3-10 μM). The concentrations of these compounds were selected based on our previous studies evaluating effects of simvastatin and resveratrol on ovarian theca-interstitial cell steroidogenesis [10,21]. All cultures were carried out in the presence of LH (5 ng/ml). All above chemicals were purchased from Sigma Chemical Co. (St. Louis, MO) except for LH, which was obtained from the National Hormone & Pituitary Program at the Harbor-UCLA Medical Center (Torrance, CA). Each experiment was repeated three times with four replicates in each experiment.

### Total RNA isolation and quantitative real-time PCR

Total RNA was isolated using the MagMAX-96 Total RNA Isolation Kit (Applied Biosystems, Foster City, CA) and the KingFisher robot (Thermo Scientific, Vantaa, Finland). Reverse transcription of total RNA to cDNA was performed using High Capacity cDNA Reverse Transcription Kit for RT-PCR (Applied Biosystems, CA).

Quantitative real-time PCR reactions were performed in triplicate using the ABI 7300 Real-time PCR System (Applied Biosystems, Foster City, CA) and 2X SYBR Green PCR Master Mix (Applied Biosystem, Warrington, UK). Data were analyzed using SDS 1.4 software (Applied Biosystems). The relative amount of target mRNA was expressed as a ratio normalized to hypoxanthine phosphoribosyltransferase (*Hprt*). The primer sequences were as described in Table 1.

**Table 1:** Primers for rat Hprt, Star, Cyp11a, Hsd3b1 and Cyp17a1

**Gene**	**Primer sequence**
*Hprt*	Forward: 5*'*-TTG TTG GAT ATG CCC TTG ACT-3*'*
	Reverse: 5*'*-CCG CTG TCT TTT AGG CTT TG-3*'*
*Star*	Forward: 5*'*- GCC TGA GCA AAG CGG TGT C-3
	Reverse: 5*'*- CTG GCG AAC TCT ATC TGG GTC TGT-3*'*
*Cyp11a1*	Forward: 5*'*- GCT GGA AGG TGT AGC TCA GG-3*'*
	Reverse: 5*'*- CAC TGG TGT GGA ACA TCT GG-3*'*
*Hsd3b1*	Forward: 5*'*- CCA GAA ACC AAG GAG GAA T-3*'*
	Reverse: 5*'*- CCA GAA ACC AAG GAG GAA T-3*'*
*Cyp17a1*	Forward: 5*'*- ACT GAG GGT ATC GTG GAT GC-3*'*
	Reverse: 5*'*- CCG TCA GGC TGG AGA TAG AC-3*'*

### Sample preparation and processing for quantification of steroids

Each sample was directly assayed; the following extraction procedure was applied to each specimen. Each sample aliquot (300 μl) was placed in a 2.0 ml autosampler vial and spiked with 150 μl of internal standard solution, *i.e.*, androsteneione-d7 and testosterone-d3. Detection and quantitation of all analytes was accomplished using selective reaction monitoring (SRM).

Androstenedione, androsterone, progesterone and the deuterated derivative of androsteneione-d7 were obtained from Steraloids (Newport, RI), whereas testosterone-d3 was obtained from Cerillient (Round Rock, TX). Acetonitrile and methanol were HPLC grade and obtained from Burdick and Jackson (Muskesgon, MI). Acetone, isopropanol, and ammonium hydroxide were Optima grade and obtained from Fisher (St. Louis, MO). Formic acid was ACS grade and obtained from EMD (Gibbstown, NJ).

### Mass spectrometry

Simultaneous detection of androstenedione, androsterone and progesterone was achieved using a novel Turbulent Flow Chromatography HPLC-MS/MS method described in our previous study [21]. The response for androstenedione, androsterone, and progesterone were linear and gave correlation coefficients (R^2^) ≥ 0.99.

### Statistical analysis

Statistical analysis was performed using JMP 9.0 software (SAS, Cary, NC). Data are presented as the mean ± SEM. Means were compared by analysis of variance followed by post-hoc testing using Tukey’s HSD Test. When appropriate, data were logarithmically transformed. A value of P < 0.05 was considered statistically significant.

## Results

### Effect of simvastatin and resveratrol on steroidogenic enzymes gene expression

**Figure 1:**
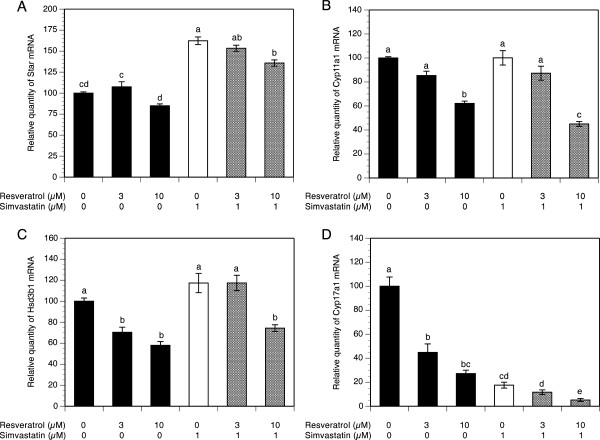
**Effect of simvastatin (1 μM) and resveratrol (3-10 μM) on mRNA expression of *Star *(A), *Cyp11a1 *(B), *Hsd3b1 *(C) and *Cyp17a1 *(D).** Theca-interstitial cells were cultured in chemically defined media supplemented with LH (5 ng/ml) for 48 h in the absence (control) or in the presence of simvastatin and/or resveratrol. Total RNA was isolated, and mRNA expression was determined using quantitative real-time PCR reactions and normalized to *Hprt* mRNA levels. Results are presented as a percentage of control. Each bar represents mean ± SEM from three independent experiments (each with N = 4). Means with no superscripts in common are significantly different (P <0.05).

To evaluate the effect of simvastatin alone and/or resveratrol on mRNA expression of the key genes regulating steroid biosynthesis pathway, theca-interstitial cells were cultured for 48 h in the absence or presence of simvastatin (1 μM) and/or resveratrol (3-10 μM). As presented in Figure 1A, resveratrol did not affect *Star* mRNA levels at any of the tested concentrations. Conversely, simvastatin induced a 1.6-fold increase in *Star* transcripts above the control level (P <0.001), whereas the addition of resveratrol to simvastatin-treated cultures had no significant effect on *Star* mRNA expression compared to the level attained with simvastatin alone, except for a modest decrease by 26% (P <0.001) at the highest concentration.

In the same experiments, resveratrol at 10 μM decreased *Cyp11a1* and *Hsd3b1* mRNA expression, respectively, by 38% and 42% (both at P <0.001), whereas simvastatin did not have any significant effect on either *Cyp11a1* or *Hsd3b1* mRNA levels. In contrast, treatment of cells with simvastatin in combination with 10 μM resveratrol decreased both *Cyp11a1* and *Hsd3b1* mRNA expression, respectively, by 55% and 43% (both at P <0.001) below the level observed with simvastatin alone (Figure 1B-C). Notably, in the presence of simvastatin, reduction of *Cyp11a1* mRNA was greater than that achieved by resveratrol alone, whereas simvastatin had no additive effect on resveratrol-induced decline of *Hsd3b1* mRNA.

The most profound resveratrol-induced inhibitory effect on mRNA expression was found in *Cyp17a1*; resveratrol at the highest concentration (10 μM) reduced *Cyp17a1* mRNA levels by 73% (P <0.001). Exposure of cells to simvastatin treatment decreased *Cyp17a1* transcripts by 82% (P <0.001) and this simvastatin-induced inhibitory effect on *Cyp17a1* mRNA expression was further enhanced to 95% (P <0.001) in the presence of 10 μM resveratrol (Figure 1D).

### Effect of simvastatin and resveratrol on steroid production

To determine the effect of simvastatin alone and/or resveratrol on steroid production, levels of progesterone, androstenedione and androsterone were evaluated in spent media using liquid chromatography-mass spectrometry. To account for both simvastatin and resveratrol potential effects on the cell number, the production of steroids was calculated per unit of protein in each individual culture well and then expressed as percentage of control cultures. Steroid levels are presented as percentage of control in order to facilitate combining of the results of three separate experiments whereby in each experiment levels of steroids in control cultures served as normalizing references. The levels of steroids in control cultures were as follows: progesterone level ranged from 5,328 pg/mg to 18,532 pg/mg (Mean = 10,484 pg/mg), androstenedione level ranged from 392 pg/mg to 3,112 pg/mg (Mean = 1,200 pg/mg) and androsterone level ranged from 657 pg/mg to 26,786 pg/mg (Mean = 11,053 pg/mg).

**Figure 2:**
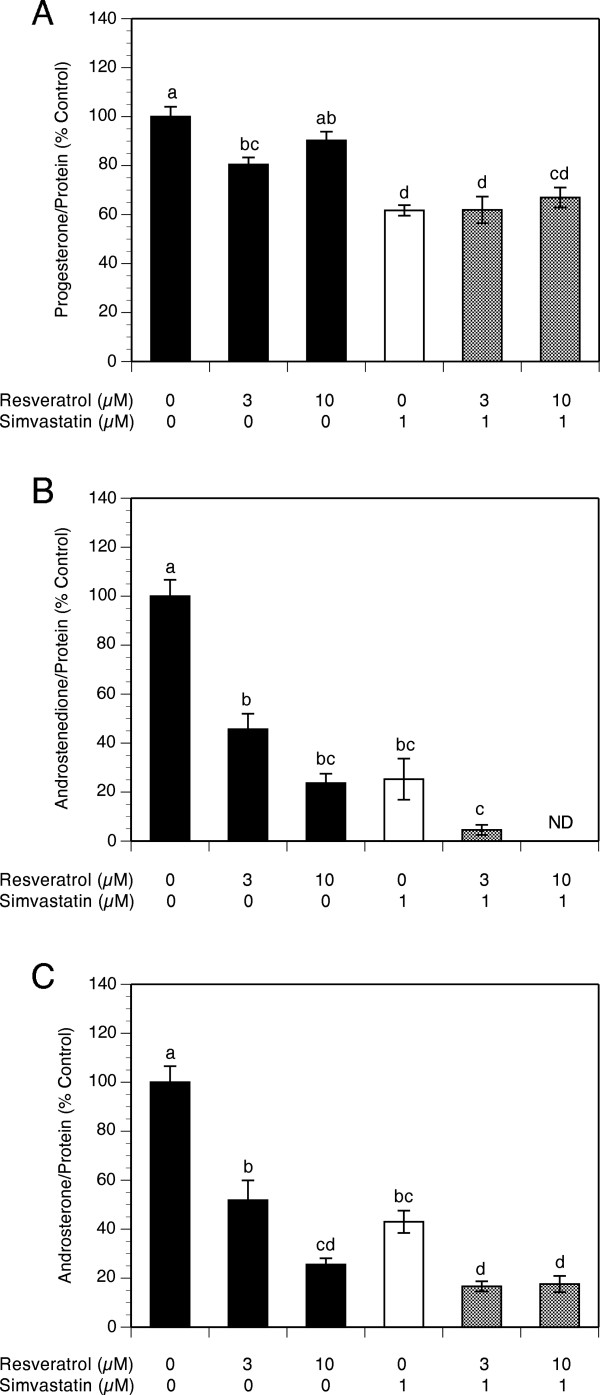
**Effect of simvastatin (1 μM) and resveratrol (3-10 μM) on steroid production by theca-interstitial cell cultures: progesterone (A), androstenedione (B) and androsterone (C).** The cells were cultured in chemically defined media supplemented with LH (5 ng/ml) for 48 h in the absence (control) or in the presence of simvastatin and/or resveratrol. Steroid levels were determined using liquid chromatography-mass spectrometry. Results are presented as a percentage of control. Each bar represents mean ± SEM from three independent experiments (each with N = 4). Means with no superscripts in common are significantly different (P <0.05).

As presented in Figure 2A, resveratrol did not affect progesterone production except for a slight decrease by 20% (P <0.01) at a concentration of 3 μM, whereas simvastatin significantly decreased progesterone production by 38% (P <0.001). The addition of resveratrol to simvastatin-treated cultures had no significant effect compared to the level observed with simvastatin alone at any of the concentrations tested.

With regard to androgen production, resveratrol induced a concentration-dependent inhibitory effect: at the highest concentration (10 μM) androstenedione levels decreased by 76% (P <0.01). Simvastatin alone inhibited androstenedione production by 83% (P <0.001) and this effect was enhanced by the addition of resveratrol whereby a combination of simvastatin and resveratrol at 10 μM reduced androstenedione to non-detectable levels (Figure 2B).

In a similar fashion, resveratrol induced a concentration-dependent decrease in androsterone levels by up to 76% at 10 μM resveratrol. Simvastatin decreased androsterone production by 57% (P <0.001), whereas the addition of 10 μM resveratrol potentiated simvastatin-induced inhibition of androsterone levels by additional 25% (P <0.001) compared to the level attained with simvastatin alone (Figure 2C).

## Discussion

This study demonstrates that in cultures of theca-interstitial cells: 1) both resveratrol and simvastatin inhibit androgen production; 2) the simvastatin-induced decrease in androgen levels is enhanced by the addition of resveratrol; 3) the combination of simvastatin and resveratrol decreases mRNA levels of key steroidogenic genes compared to simvastatin alone with particularly profound inhibition of *Cyp17a1* mRNA expression.

The novel finding of the present study is the potentiating effect of resveratrol on simvastatin-induced inhibition of steroidogenesis indicating that these compounds may exert complementary actions on mechanisms regulating ovarian steroidogenesis. The mevalonate pathway is an important cellular metabolic pathway that provides cells with diverse molecules such as cholesterol and substrates of isoprenylation: farnesyl pyrophosphate (FPP) and geranylgeranyl pyrophosphate (GGPP), which play crucial roles in cell functions [23]. Isoprenylation consists of the attachment of lipophilic FPP (farnesylation) or GGPP (geranylation) to the carboxyl terminus of proteins, regulating the function of several small guanine triphosphatases, such as Ras. Once this membrane associated GTPase has been activated, it recruits the serine/threonine kinase Raf and facilitate its activation. Then, Raf phosphorylates and stimulates the downstream kinase MEK, which in turn exhibits a serine/threonine and tyrosine kinase activity, resulting in the phosphorylation and activation of the extracellular signal-regulated kinase 1/2 (Erk1/2). The Ras-Raf-Erk1/2 signaling pathway regulates a large array of intracellular events, such as proliferation, differentiation, stress response, apoptosis and steroidogenesis. It should be noted, however, that the role of the Erk1/2 signaling pathway in steroidogenesis is, as yet, poorly understood, due to conflicting reports demonstrating stimulation, inhibition or no effect in different steroidogenic cells [27,28]. The inhibitory effect of statins on the mevalonate pathway by inhibiting HMGCR, the rate limiting step of cholesterol synthesis, leads to decreased availability of several downstream products of the pathway, including cholesterol and isoprenoids. We speculate that simvastatin inhibits theca-cell steroidogenesis by inhibiting the isoprenylation of Ras and its subsequent activity on the Ras-Raf-Erk1/2 signaling pathway. Consistent with the above considerations, our previous *in vitro* study demonstrated that simvastatin-induced inhibitory effect on theca-interstitial cell steroidogenesis is mediated, at least in part, by mechanisms involving decreased isoprenylation [9,10]. Furthermore, we previously demonstrated that another statin, mevastatin, inhibits theca-interstitial cell proliferation by selective inhibition of basal and insulin-induced activity of the Erk1/2 pathway [29].

To date, little is known regarding the role that resveratrol plays in the modulation of the mevalonate pathway. *In vivo* studies have shown that resveratrol reduces hepatic HMGCR expression as well as activity in hamsters and mice [30,31]. In our recent *in vitro* study resveratrol inhibited both HMGCR expression and activity in rat theca-interstitial cells [23]. These effects may be cell-specific since resveratrol had no significant effect on cholesterol synthesis and HMGCR activity in a study of rat hepatocytes [32].

Effects of resveratrol on the mevalonate pathway may be relevant to some and not to other effects on the function of theca-interstitial cells. We have shown that resveratrol-induced inhibition of proliferation of rat theca-interstitial cells is due, at least partly, to reduced isoprenylation [23]. However, in our recent study on effects of resveratrol on steroidogenesis, we found that the inhibitory effects were mediated by mechanisms independent of isoprenylation [21]. Indeed, in the same study we demonstrated that resveratrol decreases phosphorylation of Akt/protein kinase B (PKB), suggesting that selective inhibition of Akt/PKB pathway activity may be involved in resveratrol-induced effects on theca cell steroidogenesis.

Thus, a combination of simvastatin and resveratrol may be blocking separate crucial cell signaling pathways, such as Ras-Raf-Erk1/2 and Akt/PKB, and hence may exert inhibitory and cumulative effects on inhibition of androgen production. Previously, these pathways have been shown to be involved in the regulation of ovarian functions such as gene expression of key steroidogenic genes [33,34]. Furthermore, cross talk between the Akt/PKB and Erk1/2 pathways had been previously described in several cell types [35]. We speculate that these mechanisms of action of statin and resveratrol on key signal transduction pathways involved in steroidogenesis may account for the potentiating effect of resveratrol on simvastatin-induced inhibition of theca-interstitial cell steroidogenesis.

One potential concern regarding resveratrol pertains to its low bioavailability. In the present study, resveratrol actions were evaluated at doses ranging from 3 to 10 μM. These doses are comparable to those used in previous studies, whereby resveratrol inhibited both proliferation and steroidogenesis at concentrations in the range from 1 to 400 μM [21,36,37]. Studies on animal models and clinical trials indicate that these concentrations are likely to be clinically relevant, since the bioavailability of resveratrol in both human and rodent models is in the micromolar range [37,38]. For example, in rodents, resveratrol treatment led to its detection in multiple organs with the highest concentration in the kidney (30 μM) and liver (25 μM) [39], whereas the concentration of trans-resveratrol in the plasma ranged from 6 to 78.1 μM [40]. In humans, in a phase I study of oral resveratrol (single doses of 0.5, 1, 2.5, or 5 g) conducted in 10 healthy volunteers, peak plasma levels of resveratrol at the highest dose were 2.4 μM, whereas peak levels of resveratrol metabolites: monoglucuronides and resveratrol-3-sulfate were 3- to 8-fold higher [41]. Therefore, the concentration of resveratrol required to inhibit steroidogenesis in the present study agrees with those used in other *in vitro* studies, indicating that resveratrol at pharmacological concentrations may be effective in reducing steroidogenesis in rat theca-interstitial cells.

In the present study we have demonstrated that a combination treatment with resveratrol and simvastatin is more effective in decreasing mRNA expression of the several genes regulating the steroid biosynthesis pathway compared to treatment using simvastatin alone. Notably, the extent of inhibition in *Cyp17a1* mRNA expression induced by combination therapy was more profound compared to the effects on other genes involved in the steroidogenic function of theca-interstitial cells. Interestingly, previous studies of other biological systems have shown that resveratrol directly inhibits expression of several members of the family of human recombinant cytochromes acting as drug-metabolizing enzymes, such as CYP1A1, CYP1A2 and CYP1B1 [42-44]. A suppression of *Cyp17a1* mRNA expression, the rate-limiting step in the androgen biosynthesis pathway, decreases the conversion of progesterone into androstenedione, leading to accumulation of progesterone and decreased biosynthesis of androgens. However, in the present study the combination treatment with resveratrol and simvastatin dramatically decreased androstenedione and androsterone production, whereas levels of progesterone did not change compared to cultures with simvastatin alone. We propose that despite profound reduction of *Cyp17a1* mRNA expression, progesterone levels did not increase due to a concomitant modest decrease in mRNA expression of other genes involved in progesterone production (*Star*, *Cyp11a1* and *Hsd3b1*).

These findings may be of clinical relevance and provide a rationale for the use of a combination therapy with resveratrol and statins in treatment of hyperandrogenic conditions such as PCOS. Notably, the presently observed effects of resveratrol in combination with statin are likely to correct the key enzymatic aberrations of steroidogenesis by theca cells in women with PCOS. These aberrations include increased expression of genes regulating the androgen biosynthesis pathway including *STAR, CYP11A1, HSD3B2* and *CYP17A1*[2-4,45] as well as overexpression of *Cyp17a1* and increased activity of 17α-hydroxylase/17, 20-lyase which contribute to increased circulating levels of 17-hydroxyprogesterone in response to gonadrotropin stimulation [6,46].

## Conclusion

In conclusion, our results demonstrate for the first time that resveratrol potentiates the effects of simvastatin on inhibition of rat theca-interstitial cell androgen production. These observations may be relevant to the development of novel therapies aimed to reduce ovarian hyperandrogenism in women with PCOS.

## Abbreviations

Akt/PKB: Serine-threonine kinase (Akt)/protein kinase B; cDNA: complementary desoxirribonucleic acid; CYP11A1: Gene encoding cholesterol side chain cleavage cytochrome P450sc; CYP17A1: Gene encoding 17α-hydroxylase/C17-20 lyase cytochrome P450c17; ERK: Extracellular-signal regulated kinase; FPP: Farnesyl pyrophosphate; GGPP: Geranylgeranyl pyrophosphate; HMG-CoA: 3-hydroxy-3-methylglutaryl coenzyme A; HMGCR: 3-hydroxy-3-methylglutaryl coenzyme A reductase; HPRT: Hypoxanthine phosphoribosyltransferase; HSD3B1: Gene encoding 3β-hydroxysteroid dehydrogenase in the rat; HSD3B2: Gene encoding 3β-hydroxysteroid dehydrogenase in human; MEK: MAPK/ERK kinase; mRNA: messenger ribonucleic acid; LC-MS: Liquid cromatography-mass spectrometry; LH: Luteinizing hormone; RT-PCR: Reverse transcription polymerase chain reaction; StAR: Gene encoding steroidogenic acute regulatory protein; PCOS: Polycystic ovary syndrome.

## Competing interests

The authors declare that they have no competing interests.

## Authors’ contributions

IO planned and ran the experiments, conducted statistical analysis and wrote the manuscript. JAV, DHW, ABC, AS and SDS assisted with experiments and reviewed the manuscript. AJD planned the experiments, supervised and contributed to data interpretation and writing the manuscript. All authors read and approved the final manuscript.
